# Anti-N-Methyl-D-Aspartate Receptor Encephalitis: A Detailed Review of the Different Psychiatric Presentations and Red Flags to Look for in Suspected Cases

**DOI:** 10.7759/cureus.15188

**Published:** 2021-05-23

**Authors:** Ghasaq K Subeh, Mehreen Lajber, Talha Patel, Jihan A Mostafa

**Affiliations:** 1 College of Medicine, University of Baghdad, Baghdad, IRQ; 2 Internal Medicine, Al-Karama Teaching Hospital, Baghdad, IRQ; 3 Internal Medicine, California Institute of Behavioral Neurosciences & Psychology, Fairfield, USA; 4 Medical Education, Bacha Khan Medical College, Mardan, PAK; 5 Medical Education, California Institute of Behavioral Neurosciences & Psychology, Fairfield, USA; 6 Emergency Department, East Lancashire Hospitals NHS Trust, Blackburn, GBR; 7 Emergency, California Institute of Behavioral Neurosciences & Psychology, Fairfield, USA; 8 Psychiatry, California Institute of Behavioral Neurosciences & Psychology, Fairfield, USA

**Keywords:** anti-nmdar encephalitis, nmda receptor, autoimmune encephalitis, psychiatric disorder, neuropsychiatry

## Abstract

Anti-N-methyl-D-aspartate receptor encephalitis is a rare autoimmune disorder that involves N-methyl-D-aspartate (NMDA) receptors. It is the most common autoimmune encephalitis, and early detection and treatment are crucial for morbidity-free recovery. Distinguishing this disorder from a primary psychiatric illness is quite challenging as this disorder classically presents with psychiatric manifestations that often resemble schizophrenic psychosis. Therefore, this review intended to scope the psychiatric manifestations this disorder could present with and dissect how they differ from primary psychiatric disorders. A PubMed database search was done. The results yielded were analyzed; eventually, 50 papers were used to review the different signs and symptoms the disease can present with, including common and rare disease presentations. Diagnostic challenges and helpful clinical clues to recognize the disorder were reviewed as well.

## Introduction and background

“Like daffodils in the early days of spring, my neurons were resprouting receptors as the winter of the illness ebbed.”

- Susannah Cahalan, Brain on Fire: My Month of Madness [1].

That’s how Susannah Cahalan finally took off slowly on the road to recovery from an illness that set off her mental and bodily faculties in a downward spiral until she lost touch with reality and her own body, only to be saved later by a late but spot-on diagnosis, anti-N-methyl-D-aspartate receptor (NMDAR) encephalitis.

First described in 2007, anti-NMDAR encephalitis is a relatively modern diagnosis [2]. In anti-NMDAR encephalitis, autoantibodies are formed against NMDA glutamate receptors [2]. NMDA receptors are a subset of excitatory receptors that bind L-glutamate or structurally similar compounds. They are distributed densely in the hippocampus, other brain regions, including the cerebral cortex, the basal ganglia, and the thalamus in moderate amounts, as well as the brainstem and spinal cord [3]. It has been hypothesized, due to the clinical resemblance seen in schizophrenia and anti-NMDAR symptomatology, that the antibody-mediated downregulation of anti-NMDAR results in reduced gamma-aminobutyric acid (GABA) release in presynaptic GABAnergeic neurons, and thus the loss of inhibition on the postsynaptic glutamatergic release, leading to excess glutamate in the prefrontal and subcortical brain regions, as well as disruption in the levels of both glutamate and dopamine neurotransmitters [4]. This correlates with the fact that the hippocampus has the highest density of NMDA receptors in the brain, and thus NMDA receptor dysfunction is associated with severe amnesia [3].

This disease affects about one out of one and a half million people yearly, and it is the most common autoimmune encephalitis. The condition is more common in women, and about 37% of presenting cases are younger than 18 years old [5]. Diagnosis is based on recognizing the clinical picture and verifying the presence of NMDA antibodies in the serum or cerebrospinal fluid (CSF) [2].

Most cases are paraneoplastic and are associated with ovarian teratoma [2]. In a study that examined the presence of NMDA receptor component epitopes in non-brain tissues, immunohistochemical examinations of normal human ovaries demonstrated the presence of NR2B component of the receptor in the cytoplasm of the oocytes, which might act as a trigger to autoimmune reactions, especially in females in the reproductive age range [6]. Herpes simplex encephalitis is also a known trigger [7], although the exact pathophysiology behind the development of autoimmune encephalitis following herpes simplex virus infection has not been completely understood. It is theorized that brain tissue destruction and exposure to some neuronal antigens trigger an immune response against NMDA receptors [7]. Another possible course is brain inflammation resulting in the activation of immune cells and generation of autoantibodies, as has been seen in illnesses such as multiple sclerosis [7,8].

In about a decade, anti-NMDAR encephalitis has changed from a rare paraneoplastic syndrome to the most common cause of non-viral encephalitis. The antibodies to the NMDA receptors bind to the extracellular domain of the glutamate receptor N1 (GluN1) subunit, resulting in receptor internalization and hypofunction [9].

The course of illness can be divided into three phases: first, the prodromal period of viral infection-like symptoms, followed a couple of weeks later by psychiatric symptoms such as aggression, sleep, and behavioral changes, and finally, neurological symptoms including convulsions, catatonia, aphasia, orofacial dyskinesia, autonomic lability, and disturbed consciousness level. With early detection and treatment, most cases recover without any neurological deficits [10].

The majority of patients have severe cognitive deficits and neuropsychiatric symptoms at the time of presentation [11]. In a study that included 108 anti-NMDA encephalitis from the time of diagnosis to recovery, it was found that 45% of the patients had residual deficits in memory and speech in addition to psychiatric deficits [12]. Another study examined nine recovered patients with anti-NMDAR encephalitis who experienced neuropsychiatric deficits during the period of illness and followed them up for a period ranging from 23 to 69 months from disease onset to assess for any remnant neuropsychological deficits following recovery. The results showed remnant cognitive deficits in eight of the patients, and the patients who received treatment earlier performed better on their neuropsychological assessment, which further emphasizes the importance of early diagnosis and treatment [13].

Because this disorder is a relatively new diagnosis, it has become mandatory to thoroughly assess psychiatric manifestations in the emergency department and consider autoimmune encephalitis as a differential diagnosis in young people presenting with acute psychotic episodes [14].

Psychiatric involvement in diagnosis and treatment is crucial; antipsychotic medications have an essential role in managing agitation and aggression [15]. As most initial presentations of anti-NMDAR encephalitis patients are psychiatric, psychiatrists, as first-line responders, should familiarize themselves with this illness. One of the studies conducted a questionnaire study on how much psychiatrists are familiar with anti-NMDAR encephalitis; 76 psychiatrists were enrolled, 48.7% of whom were not aware of the disorder, 30.3% were only familiar with the name of the disorder, and 21% knew the outline of the disorder. These results showed a statistically significant low level of familiarity of psychiatrists regarding anti-NMDAR encephalitis, thus necessitating the improvement of anti-NMDAR encephalitis awareness among practicing clinicians, especially psychiatrists [16]. Because psychiatric complaints are the usual presentation of this illness, it is mandatory for practicing clinicians, especially psychiatrists and neurologists, to become familiar with the symptoms, diagnosis, and therapy, especially as the disease is reversible with early diagnosis and treatment [10]. Hence, the purpose of this review was to address the different ways anti-NMDAR encephalitis could present. The main focus was on the psychiatric presentations and identifying some key features to help differentiate those from primary psychiatric diagnoses as anti-NMDAR encephalitis tends to manifest with psychiatric symptoms first. This review was intended to help physicians familiarize themselves with this diagnosis so that early identification, treatment, and better outcomes are achieved.

## Review

The spectrum of psychiatric presentations in anti-N-methyl-D-aspartate receptor encephalitis

The course of illness in anti-NMDAR encephalitis is classically described by a prodromal period of psychiatric symptoms, speech abnormalities, and seizures. This period is then followed by neurological complications involving movement disorders and autonomic lability, and then a prolonged period of residual deficits [17].

In a study analyzing 515 patients with anti-NMDAR encephalitis, 77% of the patients presented initially with psychiatric symptoms. The most common symptom described was agitation (59%), mainly in children. Agitation was the most common presenting feature (66%), followed by psychotic symptoms (in 54%, primarily psychotic behavior, hallucinations, and delusions mostly persecutory in nature). Catatonia was also described in 42% of adult patients and 35% of children. Overall, 33% of the patients who were exposed to antipsychotics experienced neuroleptic malignant syndrome. Affect disorders were found in 30% of cases [18]. Another study was conducted to determine the frequency and temporal association between different symptoms. In the study, out of 230 cases, the most common features were seizures (60.4%), confusion (42.6%), orofacial dyskinesia (39.1%), and mutism (37.4%). Seizures, fever, and cognitive dysfunction were one of the earliest signs to emerge. The temporal sequencing of psychiatric symptoms varied vastly between individuals [19].

Anti-N-Methyl-D-Aspartate Receptor Encephalitis and Schizophrenia

The evidence supporting NMDA receptor hypofunction in schizophrenia has already been investigated [20]. In a study that investigated the relevance of anti-NMDAR encephalitis to other psychiatric diagnoses, it was found that out of a group of 51 patients who expressed symptoms on the schizoaffective spectrum, four were antibody-positive. All antibody-positive patients were females, with mild neurological symptoms, two of whom had convulsions and two had ovarian tumors [21]. Anti-NMDAR encephalitis commonly presents with psychiatric symptoms, which can resemble those seen in schizophrenia, such as hallucinations, delusions, and catatonia [22].

Catatonia as One of the Presenting Features in N-Methyl-D-Aspartate Receptor Encephalitis

Catatonia is an essential feature in anti-NMDAR encephalitis. Therefore, it has become mandatory to recognize the signs and symptoms of catatonia and recognize catatonia in and of itself as one of the hallmarks of anti-NMDAR encephalitis to initiate prompt treatment. A retrospective study estimated the frequency of catatonia to be 32.7% in a sample of 633 anti-NMDAR encephalitis patients [23]. In another study that included a sample of 58 anti-NMDAR encephalitis patients, 70.6% had catatonia, represented mainly by mutism, staring, posturing, and immobility [24].

Catatonia is a state described by a combination of mental, motor, and behavioral signs. What follows is a description of catatonic patients with anti-NMDAR encephalitis: the patients start developing decreased levels of consciousness, ending in a catatonic-like state, alternating between akinesis and agitation, they also develop a diminished response to stimuli, with some patients displaying echolalia and poor eye contact [17]. In a longitudinal prospective study, a group of patients with symptoms suggestive of anti-NMDAR encephalitis was analyzed in two groups: positive and negative anti-NMDAR antibodies. Results showed that the presence of excited-type catatonia and delirium predicted definitive anti-NMDAR encephalitis catatonia [25].

Treatment of catatonia in these patients was directed to keep the patients safe enough to get through the immunotherapy rather than treating the catatonia itself, which is why the safest psychotropic choices were used. It is important to pay attention to this point as a significant proportion of catatonic patients had received antipsychotics before being diagnosed and admitted for anti-NMDAR encephalitis [24]. These patients tend to be sensitive to antipsychotics and are more likely to develop neuroleptic malignant syndrome [26,27]; thus, early recognition of the disorder, in addition to elevating the threshold of using antipsychotics and using from the low potency category, is necessary to minimize the risk of intolerance to the psychotropic drugs [24].

Movement Disorders in Anti-N-Methyl-D-Aspartate Receptor Encephalitis

The most common phenotypes of movement disorders in anti-NMDA encephalitis can be categorized as stereotypies, motor perseveration, and orofacial dyskinesia [28,29]. Choreoathetoid movements are more commonly seen in those younger than 10 years old than those older than 18 years old [30].

Presentation With Affective Symptoms

There have been case reports of first presentations with mania [31]. One characteristic feature to look for when considering anti-NMDA encephalitis is delirious mania. In one study including 79 patients, delirious mania was seen in 20 (25.3%) patients. A significant association was observed between delirious mania and catatonia, psychomotor agitation, disinhibition and impulsivity, and delusions of grandiosity. The incidence of electroencephalogram (EEG) abnormalities was lower in cases of delirious mania, there was an absence of the classic extreme delta brush, and shorter hospital stays in cases of delirious mania [32].

Narcolepsy and Hypersomnia in Anti-N-Methyl-D-Aspartate Receptor Encephalitis

In a systematic review that described a group of anti-NMDAR encephalitis cases, a group of patients experienced narcolepsy with severe psychosis. One of the cases was a 60-year-old Parkinson’s patient with narcolepsy presenting with a severe psychotic episode that eventually required electroconvulsive therapy (ECT); anti-NMDAR antibodies were detected in retrospect. The other two cases presented with narcolepsy and severe psychosis (hallucinations and delusions), for which antipsychotics and ECT were required. Interestingly enough, another group of 10 narcoleptic patients tested for antibodies, two of whom turned out positive (one of the patients did not show psychotic features) [21].

Anti-N-Methyl-D-Aspartate Encephalitis as a Reversible Cause of Cognitive Decline

Only a small proportion of patients presenting with progressive cognitive decline suffer from reversible causes; however, all cases should be considered treatable until proven otherwise. Some of the clinical evidence to support the diagnosis of immune-mediated dementias is the triad of either catatonia or seizures plus panic and sleepiness. If found, antibody assessment is recommended before initiating a management plan. Clinical evaluation using this triad has shown that NMDA patients show panic, sleepiness, and catatonia, with or without seizures. The triad was statistically significant in differentiating degenerative and autoimmune dementias. If this triad is present, the antibodies should be evaluated first to shorten the delay in diagnosis, which is an easy bedside clue to suspect reversible dementias [33].

Anti-N-Methyl-D-Aspartate Receptor Encephalitis and Alcohol Abuse

There have been case reports of patients whose alcohol abuse problems led to an anti-NMDAR encephalitis diagnosis. A case report described a 36-year-old male alcoholic patient who presented with acute psychiatric symptoms with no abnormalities on neuroimaging and no response to stopping alcohol and symptomatic treatment. The disease progressed into developing seizures and unconsciousness, which necessitated further investigations. CSF analysis revealed anti-NMDAR antibodies, and thus the diagnosis of autoimmune encephalitis was made, and immunoglobulin therapy was initiated, after which symptoms dramatically improved. This case report suggests that autoimmune encephalitis should be considered in cases of chronic alcohol abuse when presenting with acute psychiatric symptoms. Hence, although the diagnosis is stirred in the direction of alcoholism, one should also keep anti-NMDAR encephalitis in mind [34].

Although anti-NMDAR encephalitis in cases with substance use disorder is rare, there has been a case report of a teenage girl abusing cannabis who was diagnosed with anti-NMDAR encephalitis [35].

Clinical diagnostic pearls and red flags to look for in anti-N-methyl-D-aspartate receptor encephalitis

In a systematic review of anti-NMDAR encephalitis cases, some psychiatric features were noted to be atypical to a primary psychiatric illness, such as severe agitation, speech defects, and catatonia. These are indicative of an organic rather than a primary psychiatric pathology [23]. Anti-NMDAR encephalitis patients tend to be more sensitive to antipsychotics. This was confirmed in a cohort study of 45 anti-NMDAR encephalitis patients, in which 21 (47%) patients were diagnosed with antipsychotic intolerance [26].

In cases with psychosis as a first presentation, anti-NMDAR encephalitis should be considered even in the absence of additional neurological manifestations. One of the case reports described an adolescent female with first-episode isolated psychosis as the only manifestation to be diagnosed later with anti-NMDAR encephalitis as a paraneoplastic disorder due to an ovarian teratoma [36].

Some of the things to look for when considering functional versus organic causes of psychosis are cognitive impairment that is severe relative to other domains, and patients with anti-NMDAR encephalitis tend to be more disturbed by their thought and behavioral disorders as opposed to full-blown delusions and hallucinations in their psychiatric counterparts [37,38]. A case report described a young 34-year-old female patient with a history of relapsing psychosis who presented with behavioral changes and cognitive impairment over 15 months, and finally, a psychotic episode that caused her most recent admission. Initial investigations and CSF analysis were normal, and the patient was treated with antipsychotics alone. Later, investigations revealed NMDA receptor antibodies. The patient’s psychotic episode resolved, and the associated cognitive and mood impairment improved after immunotherapy. The takeaway message from this case is that it is crucial to consider anti-NMDAR encephalitis in the list of differentials in cases where psychosis is associated with cognitive impairment, even in the light of a previous psychiatric diagnosis [39]. Approximately one in eight adults with anti-NMDAR encephalitis who have been inappropriately admitted to the psychiatric unit turns out to have had prior psychiatric episodes that were most likely caused by anti-NMDAR encephalitis [26].

As most anti-NMDAR encephalitis cases typically present with psychiatric symptoms preceding the development of the full-blown illness, it has become increasingly important to recognize the atypical variants of this illness in which psychotic symptoms predominate and are sometimes confused with schizophrenia. There is a good response to early immunotherapy, and about two-thirds of cases recover completely [40]. Spontaneous remission is a possible scenario in those with anti-NMDAR encephalitis [41]. Thus, it is important to keep this in mind as a differential diagnosis when assessing patients with a history of previous psychosis who now have features suggestive of systemic or limbic involvement. The true prevalence of anti-NMDAR encephalitis may be underestimated because of how cases can sometimes go unrecognized [39].

Although rare, anti-NMDAR encephalitis course of illness can include psychiatric features only. A study that investigated this possibility found three NMDAR antibody-positive cases out of 46 who presented with psychiatric manifestations only and fulfilled the diagnostic criteria of schizophrenia. There was also one case of an anti-NMDAR antibody patient with psychiatric symptoms only who responded well to immunotherapy [42]. In contrast to patients with schizophrenia who lose insight into their illness, patients with anti-NMDAR encephalitis tend to retain some insight into their illness despite the psychosis [43].

In addition to the positive psychotic and classic encephalitis symptoms seen in anti-NMDAR encephalitis, marked negative symptoms such as flat affect, decreased rapport, emotional withdrawal, and motor retardation have been described in some cases, same as those described in cases of functional psychoses such as schizophrenia. Hence, despite the fact that these symptoms are not as common in organic encephalitis, their presence should not distract from the possibility of such a diagnosis, especially in cases where negative symptoms such as alogia and distractibility are evident, as these are much less common in primary psychiatric illnesses and should alert toward an organic cause [37,38].

Some of the features to look for when trying to differentiate anti-NMDAR encephalitis from primary psychiatric disorders include the temporal course of illness as autoimmune encephalitis emerges over a relatively short period (days to weeks) compared to months and years in mental illnesses [17]. However, in atypical variants of the disease, the progression of neuropsychiatric symptoms can take longer than usual and should raise the index of suspicion for something other than a primary psychiatric disorder; as has been described in a case report of a 32-year-old female who presented with a depressive episode lasting for four months initially, followed by a subacute course of neurological manifestations which were to be attributed later to anti-NMDAR encephalitis [44].

New onset of seizures on the background of behavioral symptoms should alert toward anti-NMDA encephalitis. EEG findings include the characteristic “extreme delta brush” in up to one-third of adult cases [45]. Clinicians should consider anti-NMDAR encephalitis when new psychiatric symptoms are preceded by a period of viral illness, seizures, or fever, or when the presenting psychiatric features are unusual such as non-verbal auditory hallucinations [19]. Patients with anti-NMDAR encephalitis may have a positive psychiatric family history, making the diagnosis even more challenging [46].

It is important to keep in mind anti-NMDAR encephalitis when seeing young patients in the emergency department with acute-onset psychotic features, especially when most anti-NMDAR encephalitis patients get admitted to psychiatric wards and get started on treatment before being diagnosed. Hence, in young patients who present with features of plausible anti-NMDAR encephalitis with a negative initial autoimmune screen, the next set of orders should be either CSF testing for pleocytosis, oligoclonal bands, or an EEG. If either of these tests comes back with abnormal findings, anti-NMDAR encephalitis diagnosis could be established, and treatment can be commenced without any further delay [14,47].

**Figure 1:**
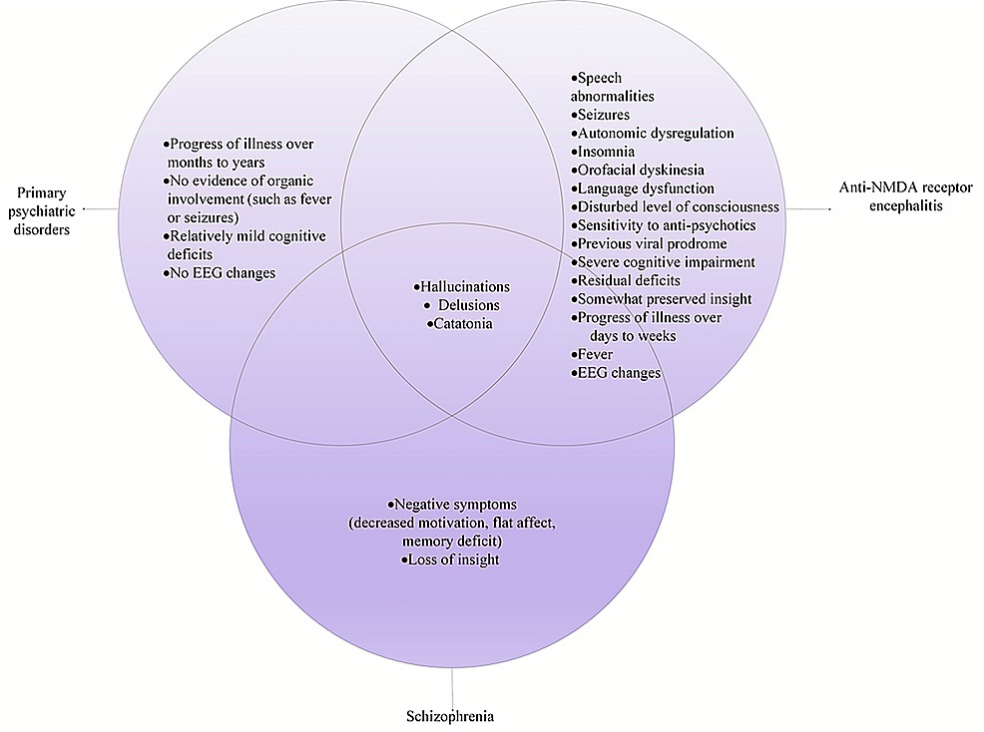
Contrast in the signs and symptoms of anti-NMDAR encephalitis, schizophrenia, and other primary psychiatric disorders. EEG: electroencephalogram; NMDA: N-methyl-D-aspartate

Figure 1 demonstrates the contrast seen clinically in anti-NMDAR encephalitis as opposed to schizophrenia and other primary psychiatric disorders.

Potential Problems With Making The Diagnosis of Anti-N-Methyl-D-Aspartate Receptor Encephalitis

Antibody testing only in cases of atypical psychotic features or waiting for disease progression to manifest neurologically runs the risk of late diagnosis and worse prognosis; therefore, antibody testing is recommended in any patient with an acute onset of psychosis or agitation, especially when it is accompanied by catatonia, disturbed consciousness level, or preceded by a flu-like illness. In cases where antibody testing is negative in the context of a compelling clinical history (for example, sensitivity to antipsychotics or the development of other suggestive symptoms), retesting in four weeks is recommended. Positive antibody testing in cases that are not suggestive of the diagnosis clinically needs further investigations with EEG, magnetic resonance imaging, and CSF analysis to guide treatment decisions [18,47]. Another important point to mention is that the absence of antibodies in CSF does not definitively exclude anti-NMDAR encephalitis; this is supported by the finding that the brain might act as a site for antibody immune complex precipitation that they cannot be detected [48].

The acute onset of psychiatric illness with no past psychiatric history is attributed to organic causes, such as anti-NMDAR encephalitis rather than a primary psychiatric illness, especially in light of a disturbed and changing level of consciousness, along with the neurological features such as seizures, dyskinesias, and other movement disorders. However, although rare, the presence of a history of psychiatric illness does not exclude the diagnosis of anti-NMDAR encephalitis. A case report described the case of a 25-year-old woman diagnosed with schizophrenia at the age of 18 and now presenting with an acute psychotic episode. The fact that there was a greater response of immunotherapy over antipsychotics, in addition to the identification of anti-NMDAR antibodies, confirmed the diagnosis of anti-NMDA encephalitis. This emphasizes the importance of considering this diagnosis even in cases with a long previous psychiatric history; hence, testing for specific immunoglobulin antibodies had become mandatory in patients who present with suggestive signs and symptoms such as a disturbed level of consciousness, confusion, cognitive deficit, dyskinesia, and autonomic instability, even in light of previous psychiatric diagnosis and negative findings on initial neuroimaging and antibody testing during the early stages of illness [49].

What seems to be purely psychiatric in nature could indeed be reversible, as has been described in a case report in which a patient’s signs and symptoms were attributed to a long-standing primary psychiatric disorder rather than an organic illness; in such cases, the question of whether it is anti-NMDAR encephalitis all along is still subject of further research [50].

## Conclusions

This review aimed to discuss the different ways anti-NMDAR encephalitis could present and manifest on the psychological, mainly, and the neurological spectrum of signs and symptoms. The signs and symptoms expressed by anti-NMDAR encephalitis patients can be categorized into psychosis (represented by delusions and hallucinations), catatonia, seizures, speech and movement abnormalities, and autonomic instability, in addition to cognitive dysfunction.

The temporal association between those signs and symptoms varied among cases; first presentations tend to overlap with psychiatric diagnoses, especially on the schizophrenic spectrum. Catatonia is one of the disease hallmarks as well. Movement disorders are commonly seen among the anti-NMDAR encephalitis pediatric population. The autoimmune link between anti-NMDAR encephalitis and schizophrenia has been described before; antibody-positive patients go on to develop neurological manifestations later on. Anti-NMDAR encephalitis can also present with affective manifestations. Narcolepsy and hypersomnia as manifestations of anti-NMDAR encephalitis have been described, as well as the association between alcohol or drug abuse and NMDAR encephalitis. There are some clinical red flags that could be of use to make the diagnosis.

Hence, for physicians trying to familiarize themselves with this diagnosis, this is a good place to start. The review included a general scheme that covers almost all aspects of the psychiatric presentation, similarities, and differences in contrast to other differential diagnoses, in addition to clinical pearls that aid diagnosis. However, this paper did not discuss the different ways this disorder presents in pediatrics; hence, this remains an area future reviews might be more inclusive of.
